# Synthesis and evaluation of 2,4,5-trisubstitutedthiazoles as carbonic anhydrase-III inhibitors

**DOI:** 10.1080/14756366.2020.1786820

**Published:** 2020-07-08

**Authors:** Bilal A. Al-Jaidi, Pran Kishore Deb, Soha Taher Telfah, Abdel Naser Dakkah, Yazan A. Bataineh, Qutaiba Ahmed Al Khames Aga, Mohammad A. Al-dhoun, Ala’ Ali Ahmad Al-Subeihi, Haifa’a Marouf Odetallah, Sanaa K. Bardaweel, Raghuprasad Mailavaram, Katharigatta N. Venugopala, Anroop B. Nair

**Affiliations:** a Department of Pharmaceutical Sciences, Faculty of Pharmacy, Philadelphia University, Amman, Jordan; b Faculty of Pharmacy, Yarmouk University, Irbid, Jordan; c Faculty of Pharmacy, Amman Arab University, Amman, Jordan; d Department of Pharmaceutical Sciences, School of Pharmacy, University of Jordan, Amman, Jordan; e Pharmaceutical Chemistry Division, Sri Vishnu College of Pharmacy, Bhimavaram, India; f Department of Pharmaceutical Sciences, College of Clinical Pharmacy, King Faisal University, Al-Ahsa, Saudi Arabia; g Department of Biotechnology and Food Technology, Durban University of Technology, Durban, South Africa

**Keywords:** Carbonic anhydrase III inhibitors, 2-amino-5-aryl-thiazole, Hummel–Dreyer method of chromatography

## Abstract

A series of 17 compounds (**12–16 b**) with 2,4,5-trisubstitutedthiazole scaffold having 5-aryl group, 4-carboxylic acid/ester moiety, and 2-amino/amido/ureido functional groups were synthesised, characterised, and evaluated for their carbonic anhydrase (CA)-III inhibitory activities using the size exclusion Hummel–Dreyer method (HDM) of chromatography. Compound **12a** with a free amino group at the 2-position, carboxylic acid moiety at the 4-position, and a phenyl ring at the 5-position of the scaffold was found to be the most potent CA-III inhibitor (*K_i _*= 0.5 μM). The presence of a carboxylic acid group at the 4-position of the scaffold was found to be crucial for the CA-III inhibitory activity. Furthermore, replacement of the free amino group with an amide and urea group resulted in a significant reduction of activity (compounds **13c** and **14c**, *K_i_* = 174.1 and 186.2 μM, respectively). Thus, compound **12a** (2-amino-5-phenylthiazole-4-carboxylic acid) can be considered as the lead molecule for further modification and development of more potent CA-III inhibitors.

## 1. Introduction

Carbonic anhydrases (CAs, EC 4.2.1.1) are a well-known superfamily of metalloenzymes, ubiquitously present in both prokaryotes as well as eukaryotes^1^^,^^2^. CAs catalyse the reversible hydration of carbon dioxide (CO_2_) into bicarbonate (HCO_3_^−^) and proton (H^+^), thereby involved in various important physiological and pathophysiological processes including acid–base regulation, ion transport, electrolyte secretions, biosynthetic reactions, and calcifications^3^^,^^4^. There are eight genetically distinguished families of CAs, viz. *α*, *ꞵ*, *γ*, *δ*, *ζ*, *η*, *θ*, and *ι*-CAs. In human, 15 isoforms of α-CAs containing Zn(II) have been reported to date, where hCAs I-III, VII, and XIII are cytosolic isoforms, hCAs IV, IX, XII, and XIV are membrane-bound isoforms and hCAs VA and VB are mitochondrial isoforms, hCA VI is mainly secreted in saliva^5^^,^^6^. It should be noted that hCAs VIII, X, and XI are characterised as a catalytic isoenzymes^7^. A large number of potential small-molecule inhibitors have been developed and some are under current clinical trial investigations targeting hCAs as potential therapeutic agents for the treatment of various diseases including diuretics, glaucoma, edema, obesity, osteoporosis, epilepsy, pain, malaria, and cancer^8–15^. It is worth mentioning that carboxylic acid group-containing environmental organic pollutants like perfluorinated alkyl substances (PFASs) do exhibit toxicity by inhibiting hCAs, which in turn lead to the disturbance of normal physiological functions^16^. However, hCA inhibitors (hCAIs) are found to be associated with various side effects mainly due to their lack of isoform specificity and off-target activity. Thus, current research efforts are focussed on the design and development of CA-specific inhibitors by using both traditional non-spectral and spectroscopic experimental approaches including recently reported innovative strategy of using narrow-bore nano-electronspray ionisation emitters in tandem with native mass spectrometry to enhance the accuracy of ligand-protein binding measurement^17–19^.

The cytosolic enzyme hCA III is specifically found to be present in liver, adipocytes, and skeletal muscle^20^. The hCA III is comparatively inefficient in catalysing CO_2_ hydration (≈300-fold less than hCA II)^21^. However, hCA III has been found to be involved in the contraction of skeletal muscle and protection of cells from reactive oxygen species (ROS) and oxidative stress^22–25^. The hCA III also facilitates the regulation of adipogenesis in relation to peroxisome proliferating-activated receptor-gamma 2 (PPAR-γ2)^26^. Furthermore, hCA III is found to be a valuable biomarker in various diseases like neuromuscular disease, sarcopenia, and hepatitis B and C infections and liver carcinoma^27–29^. It should be noted that most important classes of hCAIs contain sulphonamide (R-SO_2_NH_2_), sulphamide (R-NH_2_-SO_2_NH_2_), sulphamate (R-OSO_2_NH_2_) functional groups which are found to be insensitive towards hCA III^18^^,^^30–32^. Recently, an ureido-substituted benzene-sulphonamide (SLC 0111) has been reported to have potent hCA inhibitory activity (selective towards hCA IX/XII against hCA I and II), which is currently under Phase Ib/II clinical trials investigation for the treatment of metastatic breast cancer^33^, but, this study did not explore the selectivity profile against hCA III isoform. Thus, several heterocyclic hCA III inhibitors from natural and synthetic origin with different mechanism as compared to sulphonamides have been discovered and are currently being investigated as potential therapeutic agents for the treatment of diabetes and hyperlipidaemia^20^^,^^31^^,^^32^^,^^34^. In particular, Supuran et al. reported hCA III inhibition profile of diverse sulphonamide containing molecules including 2-aminothiadiazoles^32^. Same group of researchers further reported that most potent inhibitors of hCA-III are mainly anions like carbonate, cyanate, cyanide, thiocyanate, etc.^31^. In continuation of these observations, Alzweiri et al. reported promising hCA-III inhibitory activities of carboxylic acid containing analogues of benzoic acid and nicotinic acid^35^^,^^36^. Interestingly, vanillic acid showed more potent hCA-III inhibitory activity as compared to the acetazolamide^37^. Therefore, it was our thought of interest to synthesise and evaluate a novel series of 2,4,5-trisubstitutedthiazole derivatives with 5-aryl group, 4-carboxylic acid/ester moiety, and 2-amino/amido/ureido functional groups as possible hCA-III Inhibitors.

## 2. Materials and methods

### 2.1. Chemistry

The progress of reactions was routinely monitored by thin-layer chromatography (TLC) on silica gel plates (pre-coated Merck^®^), and spots were examined under the UV light (254 nm). Melting points were measured by open capillaries using a Stuart Scientific electro-thermal melting point apparatus (UK). FT-IR spectra were recorded on Thermo-Nicolet Avatar FT-IR (Thermo Fisher Scientific, Rockville, MD). ^1^H and ^13 ^C NMR spectra were recorded on 400 MHz Avance Ultrashield spectrometer (Bruker, Ettingen, Germany) in DMSO-*d_6_* in part per million (*δ*) using trimethylsilane as an internal standard. Mass spectra were measured in a positive ion mode using the electrospray ion trap (ESI) technique on a Bruker Apex-4 instrument (Germany).

#### 2.1.1. General methods for the synthesis of methyl 2-amino-5-substituted-4-carboxylates (12–16)

A mixture of appropriate aldehyde **1–5** (9 mM) solution in dry diethyl ether (25 mL), and methyl dichloroacetate **6** (10 mM) was kept in stirring at 0 **°**C. A pre-cooled solution of sodium methoxide (10 mM) in dry methanol was added drop wise to the aldehyde solution. After the completion of the sodium methoxide addition, the reaction mixture was kept in stirring at room temperature for 4 h. Then the reaction mixture was evaporated to dryness, and the resulted creamy residue was suspended in distilled water followed by extraction with ethyl acetate. The ethyl acetate extract was dried on anhydrous sodium sulphate and evaporated to yield the intermediate compounds **7–11** as creamy oily residue that was used for the next step without any farther purification.

Thiourea (9 mM) and the creamy residue (**7–11**) were dissolved in dry methanol and heated under reflux for 5 h and left under stirring at room temperature for 12 h. The reaction mixture was evaporated to dryness to get the yellow precipitate and suspended in water, followed by extraction with ethyl acetate. The ethyl acetate extract was dried on anhydrous sodium sulphate, and evaporated to get the solid residue. The solid residue thus obtained was recrystallized with ethyl acetate, and hexane to get the crystals of compounds **12–16** in quantitative yield (40–70%). Compounds **12** and **16** were prepared according to the reported procedure and their physical and spectral properties were found to be on par with the literature report^38^^,^^39^.

##### 2.1.1.1. Methyl-2-amino-5-(*p*-tolyl)thiazole-4-carboxylate (13)

Pale yellow crystals, yield: 54%, M.P.: 201 °C. FT-IR (ʋ cm^−1^): 3413, 3127, 1698, 1605, 1537, 1213, 996. ^1^H NMR (DMSO-*d*_6_, 400 MHz) *δ* = 2.31 (s, 3H, CH_2_), 3.62 (s, 3H, COCH_3_), 7.17 (d, 2H, NH_2_), 7.26 (m, 4H, Ar-H). ^13 ^C NMR (DMSO-*d*_6_, 400 MHz) *δ* = 20.79, 51.34, 128.14, 128.74, 129.19, 132.72, 135.22, 137.52, 162.63, 165.50. MS (ESI): *m/z* (%) = 249.1 (90) [*M* + H]^+^, 217.0 (100) [*M*-31]^+^.

##### 2.1.1.2. Methyl-2-amino-5-(4-(methylsulfonyl)phenyl)thiazole-4-carboxylate (14)

White fluffy powder, yield: 42%, M.P.: 260 °C. FT-IR: 3470, 3256, 1725, 1536, 1257, 1203, 1137. ^1^H NMR (DMSO-*d*_6_, 400 MHz) *δ* = 3.25 (s, 3H, SO_2_CH_3_), 3.66 (s, 3H, COCH_3_), 7.47 (s, 2H, NH_2_), 7.65 (d, 2H, Ar-H), 7.89 (d, 2H, Ar-H). ^13 ^C NMR (DMSO-*d*_6_, 400 MHz) *δ* = 42.48, 51.66, 126.83, 129.83, 130.11, 136.34, 136.92, 139.81, 162.41, 166.68. MS (ESI): *m/z* (%) = 312.7 (100) [*M* + H]^+^, 281.0 (90) [*M*-31]^+^.

#### 2.1.2. General methods for the synthesis of 2-amino-5-substituted-4-carboxylic acid derivatives (12a-16a)

Compounds **12–16** (2 mM) were suspended in distilled water, and 1 M NaOH solution (2 mM) was added to the reaction. The reaction mixture was warmed to 50 °C until the solution became clear and then the reaction mixture was kept in stirring at room temperature for 5 h. The reaction mixture was acidified by 1M HCl solution to pH 4 and the precipitate thus formed was collected by filtration, dried, and recrystallized with methanol and dichloromethane to get the hydrolysed compounds **12a–16a** in quantitative yield (45–80%). Compounds **12a** and **16a** were prepared according to the reported procedure and their physical and spectral properties were found to be on par with the literature report^38^.

##### 2.1.2.1. 2-Amino-5-(*p*-tolyl)thiazole-4-carboxylic acid (13a)

White fine crystals, yield: 80%, M.P.: 236 °C. FT-IR: 3302, 2945, 1694, 1585, 1297, 1284, 994. ^1^H NMR (DMSO-*d*_6_, 400 MHz) *δ* = 2.30 (s, 3H, ArCH_3_), 7.16 (bs, 2H, NH_2_), 7.26 (m, 4H, Ar-H).^13^C NMR (DMSO-*d*_6_, 400 MHz) *δ* = 20.49, 128.14, 128.74, 129.19, 132.72, 135.22, 137.52, 162.63, 165.50. MS (ESI): *m/z* (%) = 235.2 (100%) [*M* + H]^+^, 206.9 (85%) [M-17]^+^.

##### 2.1.2.2. 2-Amino-5-(4-(methylsulfonyl)phenyl)thiazole-4-carboxylic acid (14a)

Off-white crystals, yield: 74%, M.P.: 238 °C. FT-IR: 3201, 2845, 1684, 1310, 1284, 974. ^1^H NMR (DMSO-*d*_6_, 400 MHz) *δ* = 3.23 (s, 3H, SO_2_CH_3_), 7.39 (s, 2H, NH_2_), 7.65 (bs, 2H, Ar-H), 7.87 (bs, 2H, Ar-H), 12.70 (bs, 1H, COOH). ^13 ^C NMR (DMSO-*d*_6_, 400 MHz) *δ* = 43.45, 126.77, 128.44, 129.99, 136.71, 138.55, 139.56, 163.48, 166.49. MS (ESI): *m/z* (%) = 299.0 (100%) [*M* + H]^+^, 281 (40%) [M-17]^+^.

##### 2.1.2.3. 2-Amino-5-(4-chloro-3-nitrophenyl)thiazole-4-carboxylic acid (15a)

Orange crystals, yield: 45%, M.P.: 163 °C. FT-IR: 3107, 2841, 1645, 1585, 1147, 1032, 941. ^1^H NMR (DMSO-*d*_6_, 400 MHz) *δ* = 7.24 (s, 2H, NH_2_), 7.44 (m, 2H, Ar-H), 8.73 (s, 1H, Ar-H). ^13 ^C NMR (DMSO-*d*_6_, 400 MHz) *δ* = 127.76, 128.10, 131.05, 131.42, 135.21, 137.01, 154.45, 157.91, 163.61, 165.55. MS (ESI): *m/z* (%) = 301 (15%) [*M* + H]^+^, 249 (80%) [M-52]^+^.

#### 2.1.3. General methods for the synthesis of methyl-2-(4-substituted-benzamido)-5-substituted-thiazole-4-carboxylate derivatives (12 b, 14 b) and 2-(4-methoxybenzamido)-5-(4-(methylsulfonyl)phenyl)thiazole-4-carboxylic acid (14c)

Compounds **12** and **16** (2 mM) were dissolved in dry tetrahydrofuran, and triethylamine (2 mM) was added. Then 4-nitrobenzoyl chloride (2.5 mM)/4-methoxybenzoyl chloride (2.5 mM) was gradually added dropwise to the reaction mixture. The reaction mixture was kept in stirring at room temperature for 24 h. The solvent was evaporated to dryness and then suspended in water, followed by extraction with ethyl acetate. The organic layer extract was dried on anhydrous sodium sulphate and evaporated to dryness. The solid product thus obtained was recrystallized with ethyl acetate, and hexane to get the target compounds **12b** and **14b** in quantitative yields (70–77%). Compound **14b** on further hydrolysis with sodium hydroxide resulted in the formation of compound **14c**.

##### 2.1.3.1. Methyl-2-(4-nitrobenzamido)-5-substituted-thiazole-4-carboxylate (12b)

White fluffy crystals, yield: 41%, M.P.: 232 °C. FT-IR: 3399, 3115, 1707, 1674, 1556, 1296, 1256, 863. ^1^H NMR (DMSO-*d*_6_, 400 MHz) *δ* = 3.70 (s, 3H, COCH_3_), 7.44 (m, 3H, Ar-H), 7.52 (m, 2H, Ar-H), 8.34 (m, 4H, Ar-H), 13.42 (bs, 1H, CONH). ^13 ^C NMR (DMSO-*d*_6_, 400 MHz) *δ* = 51.73, 123.70, 128.36, 128.89, 129.78, 129.85, 130.09, 134.62, 137.05, 139.25, 149.83, 155.80, 162.15, 164.11. MS (ESI): *m/z* (%) = 284 (100%) [*M* + H]^+^, 351.9 (98%) [*M*-31]^+^.

##### 2.1.3.2. Methyl-2-(4-methoxybenzamido)-5-(4-(methylsulfonyl)phenyl)thiazole-4-carboxylate (14b)

Pale yellow crystals, yield: 45%, M.P.: 239 °C. FT-IR: 3251, 2918, 1722, 1652, 1513, 1202, 1089. ^1^H NMR (DMSO-*d*_6_, 400 MHz) *δ* = 3.29 (s, 3H, SO_2_CH_3_), 3.72 (s, 3H, OCH_3_), 3.84 (s, 3H, OCH_3_), 7.09 (d, 2H, Ar-H), 7.82 (d, 2H, Ar-H), 7.99 ((d, 2H, Ar-H), 8.16 (d, 2H, Ar-H),13.01 (s, 1H, CONH). ^13 ^C NMR (DMSO-*d*_6_, 400 MHz) *δ* = 43.31, 51.72, 55.46, 113.92, 123.16, 126.76, 130.31, 130.62, 135.59, 136.42, 140.51, 157.12, 161.94, 162.93, 164.88. MS (ESI): *m/z* (%) = 447 (100%) [*M* + H]^+^.

##### 2.1.3.3. 2-(4-Methoxybenzamido)-5-(4-(methylsulfonyl)phenyl)thiazole-4-carboxylic acid (14c)

White puffy powder, yield: 44%, M.P.: 174 °C. FT-IR: 3402, 3241, 2831, 1699, 1153, 1310, 1234, 1187. ^1^H NMR (DMSO-*d*_6_, 400 MHz) *δ* = 3.29 (s, 3H, SO_2_CH_3_), 3.72 (s, 3H, ArOCH_3_), 7.09 (d, 2H, Ar-H), 7.82 (d, 2H, Ar-H), 7.99 (d, 2H, Ar-H), 8.16 (d, 2H, Ar-H), 13.04 (s, 1H, COOH). ^13 ^C NMR (DMSO-*d*_6_, 400 MHz) *δ* = 43.3, 55.46, 113.92, 123.16, 126.76, 130.31, 130.62, 135.59, 136.42, 140.51, 157.12, 161.94, 162.93, 164.88. MS (ESI): *m/z* (%) = 433 (30%) [*M* + H]^+^.

#### 2.1.4. Synthesis of methyl 5-(substituted)-2-(3-phenylureido)thiazole-4-carboxylate (13b, 14d and 16 b) and 2-(3-phenylureido)-5-(p-tolyl)thiazole-4-carboxylic acid (13c)

Compound **13/14/16** (1.5 mM) was dissolved in dry dimethyl formamide, and phenyl isocyanate (2.0 mM) was added to the reaction. The reaction mixture was stirred at room temperature for 24 h. Solvent was evaporated to dryness, and the solid residue thus obtained was recrystallized with ethyl acetate and hexane to get the compounds **13b, 4d,** and **16b**, respectively, in quantitative yields. Compound **13b** on further hydrolysis with sodium hydroxide resulted in the formation of compound **13c**.

##### 2.1.4.1. Methyl-2-(3-phenylureido)-5-(*p*-tolyl)thiazole-4-carboxylate (13b)

Orange crystals, yield: 39%, M.P.: 147 °C. FT-IR: 3278, 2949, 1695, 1538, 1244, 1207, 996. ^1^H NMR (DMSO-*d*_6_, 400 MHz) *δ* = 2.38 (s, 3H, ArCH_3_), 3.67 (s. 3H, COCH_3_), 7.22 (d, 2H, Ar-H), 7.31 (m, 4H, Ar-H), 7.46 (d, 3H, Ar-H), 9.07 (s, 1H, CONH), 10.89 (s, 1H, CONH). ^13 ^C NMR (DMSO-*d*_6_, 400 MHz) *δ* = 16.02, 46.81, 113.37, 114.09, 118.22, 122.66, 123.97, 124.11, 129.28, 133.40, 133.51, 134.90, 146.91, 151.66, 157.62. MS (ESI): *m/z* (%) = 368 (100%) [*M* + H]^+^, 336 (35%) [*M*-31]^+^.

##### 2.1.4.2. Methyl 5-(4-(methylsulfonyl)phenyl)-2-(3-phenylureido)thiazole-4-carboxylate (14d)

White crystals, yield: 59%, M.P.: 143 °C. FT-IR: 3267, 3035, 1716, 1622, 1502, 1277, 1145, 961. ^1^H NMR (DMSO-*d*_6_, 400 MHz) *δ* = 3.25 (3H, s, SO_s_CH_3_), 3.66 (s, 3H, COCH_3_), 7.09 (d, 2H, Ar-H), 7.82 (d, 2H, Ar-H), 7.99 (d, 2H, Ar-H), 8.16 (d, 2H, Ar-H), 9.07 (s, 1H, CONH), 10.89 (s, 1H, CONH). ^13 ^C NMR (DMSO-*d*_6_, 400 MHz) *δ* = 43.31, 51.72, 55.46, 113.92, 123.16, 126.76, 130.31, 130.62, 135.59, 136.42, 140.51, 157.12, 161.94, 162.93, 164.88. MS (ESI): *m/z* (%) = 432 (55%) [*M* + H]^+^.

##### 2.1.4.3. Methyl-5-benzyl-2-(3-phenylureido)thiazole-4-carboxylate (16b)

Off-white powder, yield: 33%, M.P.: 226 °C. FT-IR: 3267, 3025, 1690, 1536, 1238, 1213, 900.16. ^1^H NMR (DMSO-*d*_6_, 400 MHz) *δ* = 3.79 (s. 3H, COCH_3_), 4.43 (Ar-CH_2_), 7.28 (m, 7H, Ar-H), 7.41 (m, 3H, Ar-H), 8.91 (s, 1H, CONH), 10.67 (s, 1H, CONH). ^13 ^C NMR (DMSO-*d*_6_, 400 MHz) *δ* = 27.20, 46.84, 114.05, 118.15, 121.87, 123.71, 123.83, 124.07, 129.83, 133.50, 134.90, 136.66, 146.84, 150.99, 157.71. MS (ESI): *m/z* (%) = 368 (100%) [*M* + H]^+^.

##### 2.1.4.4. 2-(3-Phenylureido)-5-(*p*-tolyl)thiazole-4-carboxylic acid (13c)

White puffy powder, yield: 54%, M.P.: 226 °C. FT-IR: 3395, 2947, 1685, 1538, 1207, 1187, 996. ^1^H NMR (DMSO-*d*_6_, 400 MHz) *δ* =2.32 (s, 3H, ArCH_3_), 7.00 (t, 1H, Ar-H), 7.20 (d, 2H, Ar-H), 7.28 (t, 2H, Ar-H), 7.35 (d, 1H, Ar-H), 7.47 (d, 2H, Ar-H), 10.34 (bs, 1H, CONH), 11.17 (bs, 1H, COOH). ^13 ^C NMR (DMSO-*d*_6_, 400 MHz) *δ* = 28.01, 113.95, 118.04, 122.21, 123.71, 123.63, 124.42, 129.83, 133.50, 135.19, 136.66, 145.84, 150.84, 160.11. MS (ESI): *m/z* (%) = 353.9 (30%) [*M* + H]^+^, 307 (100%) [M-46]^+^.

### 2.2. Carbonic anhydrase – III inhibition assay

#### 2.2.1. Instrumentation

HPLC screening was conducted on the LC-2010A HT HPLC system, coupled with column temperature controller, degasser, auto sampler, and isocratic elution system, LC-solution software (to calculate peak area), Shimadzu Corporation, Kyoto, Japan. Back pressure was maintained around 6 MPA. A Phenomenex BioSep-SEC s2000, 300 × 7.8 mm column was used for size exclusion chromatography.

#### 2.2.2. Materials

Bovine carbonic anhydrase, isozyme III (Biovar Ltd, Armenia) was stored at 0 °C in refrigerator until use. Enzyme was left for few minutes at room temperature before analysis. HPLC-grade methanol and acetonitrile (Fisher Scientific, Loughborough, UK) were used without further purification. NaH_2_PO_4_.H_2_O (Gainland, Deeside, UK), HCl (Carlo Erba, Italy), NaOH (Lonver, UK), and deionised water were used for the preparation of mobile phase. All the required chemicals for the synthesis of target compounds (**12–16b**) were procured either from Fisher Scientific, Loughborough, UK or Sigma-Aldrich, St. Louis, MO.

#### 2.2.3. Mobile phase and sample preparation

Each target compounds (**12–16b**) was dissolved in a mixture of acetonitrile: phosphate buffer, pH 6.5 (1:9 v/v) to obtain a final concentration of 0.24 mM. Subsequently, filtration and degassing by sonication were conducted for the mobile phase. Three different enzyme (CA-III) concentrations of 1.065, 1.7, and 1.92 mM were prepared from the stock solution (containing 0.865 mg in 10 mL of distilled water) by subsequent dilution method and injected separately in each run in the chromatographic system. All samples were used in duplicate. Vanillic acid was used as a positive control, and toluene was used as a negative control.

The mobile phase used for analysis was chosen according to the optimised conditions obtained, where 10% of organic modifier, 30 mM phosphate buffer (pH = 6.5) and 37 °C temperature was used. Column was pre-equilibrated with the mobile phase for 50 min. The flow rate of mobile phase was maintained at 1 mL/min during the run time. A wavelength of 230 nm (*λ*_max_) was selected for the analysis of all the tested compounds.

#### 2.2.4. Optimisation of HPLC ligand–enzyme interaction

The interaction between the enzyme and ligand in the Hummel–Dreyer method (HDM) was optimised using vanillic acid as a reference standard, while changing four experimental conditions: buffer concentration, pH of the mobile phase, temperature, and proportion of organic modifier, respectively. In order to study the effect of a particular chromatographic condition, one of the four conditions was varied, and the other three conditions were kept constant. Each reading represents the average of duplicate measurements and the one achieving the optimum interaction between the enzyme and ligand was used for further consideration.

##### 2.2.4.1. Optimisation of buffer concentration

The effect of buffer concentration was investigated by systematically increasing the concentration from 0.2 to 35 mM. The column was thermostatted at 37 °C throughout the chromatographic run. The mobile phase composition was 10% acetonitrile in phosphate buffer with pH = 6. Optimum buffer concentration was considered to be 10 mM.

##### 2.2.4.2. Optimisation of mobile phase pH

The effect of pH was investigated by increasing pH in 0.5 increment rate starting from 5.5 to 7.5. Column pH toleration was taken into consideration and the maximum area of negative peak was chosen accordingly. The column was thermostatted at 37 °C. Optimum interaction was found at pH = 6.5.

##### 2.2.4.3. Optimisation of organic solvent percent

The effect of organic modifier proportion was investigated by increasing the volume fraction of acetonitrile in the mobile phase by an increment of 5. Starting from 5 to 40%, the maximum area of negative peak was chosen accordingly, indicating higher interaction between enzyme and ligand. The column was thermostatted at 37 °C. Optimum interaction was observed by using 10% acetonitrile at a buffer concentration of 10 mM, and pH 6.5.

##### 2.2.4.4. Optimisation of temperature

The column temperature was gradually increased from 25  to 50 °C (which is the maximum tolerated temperature by the column). Selected temperatures were: 25, 30, 35, 36, 37, 38, 40, 45, and 50 °C. There was a sharp increase in the interaction by increasing temperature, however, increasing temperature could affect the column longevity, thereby 37 °C was chosen as the optimum temperature.

## 3. Result and discussion

### 3.1. Chemistry

**Scheme 1.**
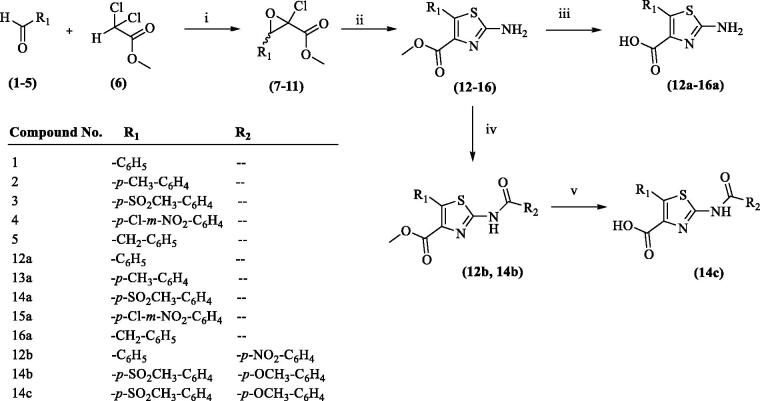
Synthesis of 2-amino-5-substituted-4-carboxylic acid derivatives (12a–16a) and 2-amido-5-substituted-4-carboxylate/carboxylic acid derivatives (12 b, 14 b and 14c). Reagents and conditions: (i) NaOCH_3_, ether, 0 °C, stirring, 5 h; (ii) thiourea, methanol, reflux, 3 h; (iii) NaOH, H_2_O, stirring, 5 h; (iv) R_2_COCl, tretrahydrofuran (THF), stirring, room temperature, 5 h; (v) NaOH, H_2_O, stirring, overnight.

**Scheme 2.**
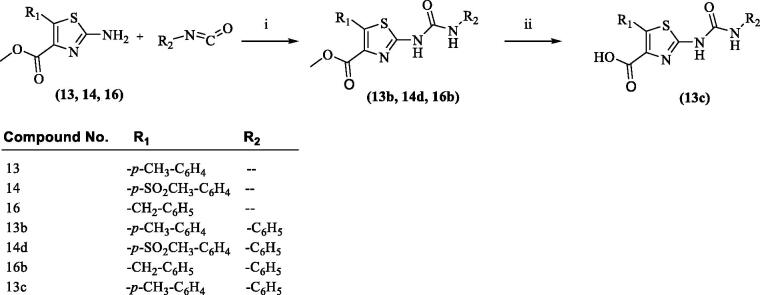
Synthesis of 2-uriedothiazole derivatives (**13b, 14d, 16b, 13c**). Reagents and conditions: (i) DMF, room temperature, stirring, 24 h; (ii) NaOH, H_2_O, room temperature, stirring, overnight.

A series of 17 compounds with 2,4,5-trisubstitutedthiazole scaffold were synthesised by following Schemes 1 and 2. Compounds **7–11** were synthesised following well known Drazen’s reaction procedure^40^ by treating various aldehydes (**1–5**) with methyldichloroacetate (**6**) in dry ether under basic condition using sodium methoxide. Compounds **7–11** on further reaction with thiourea under reflux condition in methanol resulted in the formation of methyl 2-amino-5-substituted-4-carboxylate derivatives (**12–16**). Hydrolysis of compounds **12–16** with aqueous sodium hydroxide solution resulted in the formation of 2-amino-5-substituted-4-carboxylic acid derivatives (**12a–16a**). Compounds **12** and **14** on reaction with 4-nitrobenzoyl chloride and 4-methoxybenzoyl chloride, respectively, at room temperature under basic medium (triethylamine) resulted in the formation of compounds **12b**, and **14b**. Compound **14b** on hydrolysis with sodium hydroxide resulted in the formation of compound **14c**. Compounds **12, 16, 12a,** and **16a** were prepared according to the reported procedures and their physical and spectral properties were found to be on par with the literature report^38^.

Compounds **13, 14,** and **16** on further reaction with phenylisocyanate at room temperature under stirring resulted in the formation of compounds **13b, 14d,** and **16b**, respectively. Hydrolysis of compounds **13b** with sodium hydroxide resulted in the formation of compound **13c**.

### 3.2. Carbonic anhydrase – III inhibition assay

CA-III inhibitory activities of all the 17 target compounds (**12–16b**) as shown in Table 1 were carried out by using size exclusion HDM chromatography^35–37^. The HDM chromatography was preferred over colorimetric assay of CA inhibitors, due to the susceptibility of the colorimetric methods to the intrinsic acidic or alkaline properties of analytes (inhibitors), including the lower catalytic activity of CA-III in CO_2_ hydration. Moreover, in HDM chromatographic method, the dilution effect of the mobile phase not only provides comparatively larger space for interaction between the macromolecule (CA-III) and analytes, but also lowers the probability of self-aggregation of macromolecules^35–37^^,^^41^. The HDM was further optimised prior to use, where mobile phase was buffered to maintain the pH = 6.5 to mimic the physiological condition and to minimise the intrinsic properties of analytes. Moreover, acetonitrile (10%) was used to facilitate the elution of analytes (inhibitors) from the hydrophobic column as well as to prevent the adhesion of macromolecule to the stationary phase of the column. A constant optimised temperature of 37 °C was maintained throughout the chromatographic run, as the increase in temperature could not only influence the protein-ligand interaction but also affect the longevity of the column.

**Table 1.** Carbonic anhydrase (CA-III) inhibitory activities of compounds 12–16b.


Compound no.	R_1_	R_2_	K*_i_* (µM)
			CA-III
12	-C_6_H_5_	H	>500
13	-*p*-CH_3_-C_6_H_4_	H	>500
14	-*p*-SO_2_CH_3_-C_6_H_4_	H	>500
15	-*p*-Cl-*m*-NO_2_-C_6_H_4_	H	>500
16	-CH_2_-C_6_H_5_	H	>500
12a	-C_6_H_5_	H	0.5
13a	-*p*-CH_3_-C_6_H_4_	H	18.9
14a	-*p*-SO_2_CH_3_-C_6_H_4_	H	86.6
15a	-*p*-Cl-*m*-NO_2_-C_6_H_4_	H	81.2
16a	-CH_2_-C_6_H_5_	H	>500
12b	-C_6_H_5_	-*p*-OCH_3_-C_6_H_4_CO	>500
13b	-*p*-CH_3_-C_6_H_4_	-C_6_H_5_	>500
14b	-*p*-SO_2_CH_3_-C_6_H_4_	-*p*-NO_2_-C_6_H_4_CO	>500
16b	-CH_2_-C_6_H_5_	-C_6_H_5_	>500
13c	-*p*-CH_3_-C_6_H_4_	-C_6_H_5_	174.1
14c	-*p*-SO_2_CH_3_-C_6_H_4_	-*p*-NO_2_-C_6_H_4_CO	186.2
14d	-*p*-SO_2_CH_3_-C_6_H_4_	-C_6_H_5_	>500
Vanillic acid	–	–	6.8

**Figure 1.**
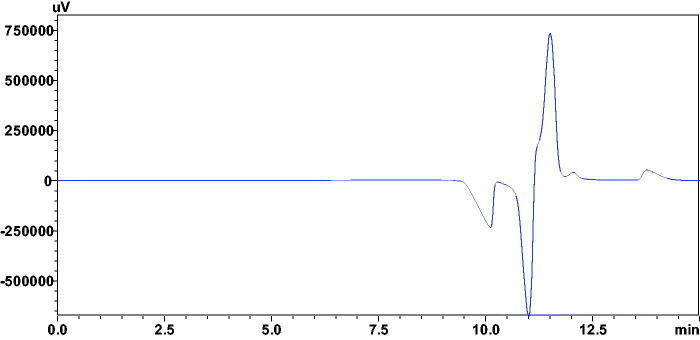
Chromatogram showing vacancy peak (V.P), resulted after the injection of 1.7 mM CA-III solution, with mobile phase containing 0.24 mM of the compound 12a.

The chromatographic analysis by HDM is basically based on the measurement of the intensity of the formation of vacancy (negative) peak due to the subtraction of CA-III bound portion of analytes from the mobile phase. This method is also independent of the enzyme catalytic activity, thereby considered as more sensitive method for the estimation of inhibitory activities of analytes as compared to the colorimetric assay^35–37^^,^^41^. Thus, weak inhibitors created weak vacancy peaks independent of the enzyme concentrations. In case of potent inhibitors, intense vacancy peaks directly proportional to the low concentrations of CA-III was observed, followed by a steady state at higher concentrations of CA-III solutions (Figure 1).

Different concentrations of CA-III were injected in the chromatographic system containing a constant concentration (0.24 mM) of analytes **(12a–16a)** in the mobile phase. HPLC quantification was generated with Shimadzu’s LC-solution software coupled with Shimadzu instrument. Absorbance (A) as a response in terms of detector signal is obtained from the negative-peak area determination. This response was utilised to measure the concentration or amount of the ligand bound with the CA-III enzyme [*I_B_*], by dividing the area obtained for absorbance of negative peak by absorptivity (a) of the standard ligand with known concentration according to Beer–Lambert law:

(1) A = aBC

where *A* = absorbance, *a* = absorptivity, *B* = cell bath length, and *C* = concentration.

The absorptivity (*a*) of the standard was then deduced from the known concentration and absorbance of the standard using Beer–Lambert law. The concentration of ligand bound to CA-III (which represents the amount of ligand removed from the stationary phase as a result of binding to the enzyme) was measured again using the same equation.

The inhibition constant (*K_i_*) values were calculated according to the previously reported^37^ following equation (2):

(2) $$K_{i}=\frac{\left[I]{(E-I}_{B}\right)}{I_{B})}\;$$

where [*I*] is the concentration of the analyte in the mobile phase, which is constant (0.24 mM) for all ligands; [*I*_B_] is the bound amount of the inhibitor (mM); [*E*] is the amount of enzyme (mM) injected in HPLC.

Compounds **(12a–16a)** with a free amino group at 2-position and a carboxylic acid moiety at 4-position, having an aromatic ring at 5-position of the thiazole scaffold showed good CA-III inhibitory activity. In particular, compound **12a** with a phenyl ring at 5-position was found to be the most potent CA-III inhibitor (*K_i _*= 0.5 μM) (Figure 1). Substitution at the *para* position of the phenyl ring of the scaffold reduced the activity for compounds **13a, 14a,** and **15a** (*K_i_* = 18.9, 86.6, and 81.2 μM, respectively). However, replacing the phenyl ring with a benzyl ring completely abolished the activity for the compound **16a** (*K_i _*> 500 μM). Interestingly, compounds **12–16, 12b, 13b, 14b, 14d,** and **16b** with carboxylic ester group at 4-position did not show any activity (*K_i _*> 500 μM), indicating the importance of the carboxylic group in CA-III inhibitory activity for compounds **12a–16a** probably by inducing electrostatic interaction at the binding site of the enzyme. Further, it has been observed that in case of carboxylic acid derivatives, conversion of free amino group into amide and urea group in compounds **13c** and **14c** resulted in a significant reduction of activity (*K_i _*= 174.1 and 186.2 μM, respectively) as compared to the compounds **13a** and **14a** (*K_i_*=18.9 and 86.6 μM, respectively).

## 4. Conclusions

In this work, a series of 17 compounds (**12–16b**) with 2,4,5-trisubstitutedthiazole scaffold were synthesised and evaluated for their CA-III inhibitory activities using size exclusion HDM chromatography. Compound **12a** with a free amino group at 2-position, carboxylic acid moiety at 4-position, and a phenyl ring at 5-position of the scaffold was found to be the most potent CA-III inhibitor (*K_i _*=0.5 μM). Substitution at the *para* position of the phenyl ring of the scaffold reduced the activity in case of the compounds **13a–15a**; whereas, the replacement of phenyl ring with a bulky benzyl ring completely abolished the activity (compound **16a**, *K_i_*>500 μM). Interestingly, the presence of a carboxylic acid group at the 4-position of the scaffold was found to be of prime importance for CA-III inhibitory activity, probably by inducing electrostatic interaction at the binding site of the enzyme; as evident from compounds **12–16, 12b, 13b, 14b, 14d,** and **16b** having carboxylic ester group at 4-position with no CA-III inhibitory activities (*K_i_*>500 μM). Furthermore, replacement of the free amino group with amide and urea group resulted in a significant reduction of activity (compounds **13c** and **14c**, *K_i_*=174.1 and 186.2 μM). Thus compound **12a** can be considered as a lead molecule for further modification and development of more potent CA-III inhibitors.

## References

[1] Supuran CT, Nocentini A. Carbonic anhydrases: biochemistry and pharmacology of an evergreen pharmaceutical target. Amsterdam, Netherlands: Elsevier Science; 2019.

[2] Aspatwar A, Tolvanen ME, Ortutay C, Parkkila S. Carbonic anhydrase related proteins: molecular biology and evolution. Carbonic anhydrase: mechanism, regulation, links to disease, and industrial applications. Berlin, Germany: Springer; 2014. 10.1007/978-94-007-7359-2_8 24146378

[3] Khalifah RG. The carbon dioxide hydration activity of carbonic anhydrase i. Stop-flow kinetic studies on the native human isoenzymes b and c. J Biol Chem 1971;246:2561–73. 4994926

[4] Supuran CT. How many carbonic anhydrase inhibition mechanisms exist? J Enzym Inhib Med Chem 2016;31:345–60. 10.3109/14756366.2015.1122001 26619898

[5] D’Ambrosio K, De Simone G, Supuran CT. Human carbonic anhydrases: catalytic properties, structural features, and tissue distribution. Carbonic anhydrases as biocatalysts. Amsterdam, Netherlands: Elsevier; 2015.

[6] Supuran CT. Structure and function of carbonic anhydrases. Biochem J 2016;473:2023–32. 27407171 10.1042/BCJ20160115

[7] Supuran CT, Capasso C. Acatalytic carbonic anhydrases (cas viii, x, xi). Carbonic anhydrases as biocatalysts. Amsterdam, Netherlands: Elsevier; 2015.

[8] Supuran CT. Carbonic anhydrases: from biomedical applications of the inhibitors and activators to biotechnological use for co2 capture. Abingdon: Taylor & Francis; 2013. 10.3109/14756366.2013.761876 23379684

[9] Carta F, Supuran CT. Diuretics with carbonic anhydrase inhibitory action: a patent and literature review (2005–2013). Exp Opin Ther Patent 2013;23:681–91. 10.1517/13543776.2013.780598 23488823

[10] Supuran CT, Altamimi ASA, Carta F. Carbonic anhydrase inhibition and the management of glaucoma: a literature and patent review 2013–2019. Exp Opin Ther Patent 2019;29:781–92. 10.1080/13543776.2019.1679117 31596641

[11] De Simone G, Supuran CT. Antiobesity carbonic anhydrase inhibitors. Curr Top Med Chem 2007;7:879–84. 17504132 10.2174/156802607780636762

[12] Aggarwal M, Kondeti B, McKenna R. Anticonvulsant/antiepileptic carbonic anhydrase inhibitors: a patent review. Exp Opin Ther Patent 2013;23:717–24. 10.1517/13543776.2013.782394 23514045

[13] Supuran CT. Carbonic anhydrase inhibition and the management of neuropathic pain. Exp Rev Neurother 2016;16:961–8. 10.1080/14737175.2016.1193009 27211329

[14] Del Prete S, Vullo D, Fisher GM, et al Discovery of a new family of carbonic anhydrases in the malaria pathogen plasmodium falciparum-the η-carbonic anhydrases. Bioorg Med Chem Lett 2014;24:4389–96. 25168745 10.1016/j.bmcl.2014.08.015

[15] Supuran CT. Carbonic anhydrase inhibitors as emerging agents for the treatment and imaging of hypoxic tumors. Exp Opin Invest Drugs 2018;27:963–70. 10.1080/13543784.2018.1548608 30426805

[16] Nguyen GT, Nocentini A, Angeli A, et al. Perfluoroalkyl substances of significant environmental concern can strongly inhibit human carbonic anhydrase isozymes. Anal Chem 2020;92:4614–22. 32096628 10.1021/acs.analchem.0c00163

[17] Alterio V, Di Fiore A, D’Ambrosio K, et al. Multiple binding modes of inhibitors to carbonic anhydrases: how to design specific drugs targeting 15 different isoforms? Chem Rev 2012;112:4421–68. 22607219 10.1021/cr200176r

[18] Supuran CT. Advances in structure-based drug discovery of carbonic anhydrase inhibitors. Exp Opin Drug Discov 2017;12:61–88. 10.1080/17460441.2017.1253677 27783541

[19] Nguyen GT, Tran TN, Podgorski MN, et al Nanoscale ion emitters in native mass spectrometry for measuring Ligand-Protein Binding Affinities. ACS Central Sci 2019;5:308–18. 10.1021/acscentsci.8b00787 PMC6396573 30834319

[20] Mahon BP, McKenna R. Carbonic anhydrase iii. Carbonic anhydrases as biocatalysts. Amsterdam, Netherlands: Elsevier; 2015.

[21] Ren X, Jonsson BH, Lindskog S. Some properties of site-specific mutants of human carbonic anhydrase II having active-site residues characterizing carbonic anhydrase III. Eur J Biochem 1991;201:417–20. 1935938 10.1111/j.1432-1033.1991.tb16299.x

[22] Geers C, Gros G. Effects of carbonic anhydrase inhibitors on contraction, intracellular ph and energy-rich phosphates of rat skeletal muscle. J Physiol 1990;423:279–97. 2388152 10.1113/jphysiol.1990.sp018022 PMC1189757

[23] Räisänen SR, Lehenkari P, Tasanen M, et al. Carbonic anhydrase iii protects cells from hydrogen peroxide-induced apoptosis. FASEB J 1999;13:513–22. 10064618 10.1096/fasebj.13.3.513

[24] Wroblewski K, Spalthoff S, Zimmerman UJ, et al. The role of carbonic anhydrase in the recovery of skeletal muscle from anoxia. J Appl Physiol 2005;99:488–98. 15802363 10.1152/japplphysiol.01409.2004

[25] Staunton L, Zweyer M, Swandulla D, Ohlendieck K. Mass spectrometry-based proteomic analysis of middle-aged vs. Aged vastus lateralis reveals increased levels of carbonic anhydrase isoform 3 in senescent human skeletal muscle. Int J Mol Med 2012;30:723–33. 22797148 10.3892/ijmm.2012.1056 PMC3573712

[26] Mitterberger MC, Kim G, Rostek U, et al. Carbonic anhydrase iii regulates peroxisome proliferator-activated receptor-γ2. Exp Cell Res 2012;318:877–86. 22507175 10.1016/j.yexcr.2012.02.011 PMC3328775

[27] Heath R, Schwartz MS, Brown IR, Carter ND. Carbonic anhydrase iii in neuromuscular disorders. J Neurol Sci 1983;59:383–8. 6410007 10.1016/0022-510x(83)90023-0

[28] Dai HY, Hong CC, Liang SC, et al. Carbonic anhydrase iii promotes transformation and invasion capability in hepatoma cells through fak signaling pathway. Mol Carcinog 2008;47:956–63. 18444244 10.1002/mc.20448

[29] Elchuri S, Naeemuddin M, Sharpe O, et al. Identification of biomarkers associated with the development of hepatocellular carcinoma in cuzn superoxide dismutase deficient mice. Proteomics 2007;7:2121–9. 17514684 10.1002/pmic.200601011 PMC2729784

[30] Carta F, Supuran CT, Scozzafava A. Sulfonamides and their isosters as carbonic anhydrase inhibitors. Fut Med Chem 2014;6:1149–65. 10.4155/fmc.14.68 25078135

[31] Nishimori I, Minakuchi T, Onishi S, et al. Carbonic anhydrase inhibitors. Cloning, characterization and inhibition studies of the cytosolic isozyme iii with anions. J Enzym Inhib Med Chem 2009;24:70–6. 10.1080/14756360801907143 18618322

[32] Nishimori I, Minakuchi T, Onishi S, et al. Carbonic anhydrase inhibitors: cloning, characterization, and inhibition studies of the cytosolic isozyme iii with sulfonamides. Bioorg Med Chem 2007;15:7229–36. 17826101 10.1016/j.bmc.2007.08.037

[33] Carta F, Vullo D, Osman SM, et al. Synthesis and carbonic anhydrase inhibition of a series of slc-0111 analogs. Bioorg Med Chem 2017;25:2569–76. 28347633 10.1016/j.bmc.2017.03.027

[34] Alver A, Uçar F, Keha EE, et al. Effects of leptin and insulin on ca iii expression in rat adipose tissue. J Enzym Inhib Med Chem 2004;19:279–81. 10.1080/14756360410001720445 15500001

[35] Alzweiri M, Al-Balas Q, Al-Hiari Y. Chromatographic evaluation and qsar optimization for benzoic acid analogues against carbonic anhydrase iii. J Enzym Inhib Med Chem 2015;30:420–9. 10.3109/14756366.2014.940939 25068727

[36] Jarrar N, Alzweiri M, Al-Hiari Y, et al. Modified hummel-dreyer method and molecular modeling studies identified nicotinic acid analogues as carbonic anhydrase iii ligands. Lett Drug Design Discov 2016;13:401–10.

[37] Alzweiri M, Al‐Hiari Y. Evaluation of vanillic acid as inhibitor of carbonic anhydrase isozyme iii by using a modified Hummel-Dreyer method: approach for drug discovery. Biomed Chromatogr 2013;27:1157–61. 23605884 10.1002/bmc.2921

[38] Al-Balas Q, Anthony NG, Al-Jaidi B, et al. Identification of 2-aminothiazole-4-carboxylate derivatives active against mycobacterium tuberculosis h37rv and the β-ketoacyl-acp synthase mtfabh. PLoS One 2009;4:e5617. 19440303 10.1371/journal.pone.0005617 PMC2680598

[39] Barton A, Breukelman SP, Kaye PT, et al. The preparation of thiazole-4-and-5-carboxylates, and an infrared study of their rotational isomers. J Chem Soc Perkin Trans 1982;1:159–64.

[40] Darzens G. The general method of the synthesis of aldehydes with the help of substituted glycidic acids. Compt Rend Hebdomad Sean Acad Sci 1904;139:1214–7.

[41] Berger G, Girault G. Macromolecule-ligand binding studied by the Hummel and Dreyer method: current state of the methodology. J Chromatogr B Analyt Technol Biomed Life Sci 2003;797:51–61. 10.1016/s1570-0232(03)00482-3 14630143

